# Anti-arthritic activity of ethanol extract of *Claoxylon indicum* on Freund’s complete adjuvant-induced arthritis in mice

**DOI:** 10.1186/s12906-016-1500-7

**Published:** 2017-01-05

**Authors:** Yong Chen, Qi-wen Wang, Jian Zuo, Jian-wei Chen, Xiang Li

**Affiliations:** 1 College of Pharmacy, Nanjing University of Chinese Medicine, POB 210023 Nanjing, China; 2 Jiangsu Key Laboratory of Therapeutic Material of Chinese Medicine, POB 210023 Nanjing, China

**Keywords:** *Claoxylon indicum*, Rheumatoid arthritis (RA), Adjuvant arthritis, Inflammation

## Abstract

**Background:**

*Claoxylon indicum* Hassk. (Euphorbiaceae), named Diu Le Bang, have functions of dehumidification and relieving swelling pain, and is used as a folk medicine to treat Rheumatoid arthritis (RA), lumbocrural pain and foot edema in the south of China. The aim of the present study was to investigate the anti-arthritic activity of the ethanol extract of *Claoxylon indicum* (CIE) on mice with adjuvant induced joint arthritis.

**Methods:**

Adjuvant arthritis was induced in mice by subcutaneous injection of complete Freund’s adjuvant into the plantar surface of right hind paw. Arthritis severity was evaluated by arthritic score, hind paws oedema and spleen index, and histological examinations. Serum samples were collected for determination of malondialdehyde (MDA) and alkaline phosphatase (ALP) levels. The expression of interleukin-1 beta (IL-1β) and tumor necrosis factor alpha (TNF-α) in the specimens of knee joints was determined by standard immunohistochemical techniques.

**Results:**

CIE administration (0.4 and 0.8 g/kg) suppressed the inflammatory responses in the joints of adjuvant-induced arthritis (AIA) mice, suggested by the modulatory effects on paw swelling, hyperplasia of lymphatic tissues and synovial membrane. It also decreased the levels of MDA and ALP in serum and downregulated the expression of IL-1β and TNF-α in the arthritic joints of AIA mice.

**Conclusion:**

These results suggested that CIE possessed substantial anti-arthritic activity due to immumodepression and regulation of cytokines. CIE may be a potential candidate for the treatment of RA.

## Background

Rheumatoid arthritis (RA) is a major worldwide public health problem in which patients suffer from deformed and painful joints. As a chronic, inflammatory and systemic autoimmune disease, RA can lead to loss of function affecting more than 10 million people in China and about 1% of the general population in the world [1]. Although a number of drugs have been developed and used for the treatment of RA, such as non-steroidal anti-inflammatory drugs (NSAIDs, e.g., Nimesulide), disease-modifying anti-rheumatic drugs (DMARDs, e.g., Leflunomide) and biological drugs (e.g., Abatacept), they are too expensive, and have selective efficacy and potential unknown threat [2–4]. Traditional Chinese medicine (TCM), with low cost and less side effects, has advantages in the treatment of chronic diseases [5].

*Claoxylon indicum* Hassk. (Euphorbiaceae) (CI), the root of which is also known as Diu Le Bang (DLB) distributed in the southwest to the southeast of China, has been used to treat RA as folk medicine for a long history [6, 7]. *Securidaca inappendiculata* Hassk. (Polygalaceae) (SI) has also been used as Diu Le Bang to cure fractures and RA. Our previous research exhibited that SI possessed substantial anti-arthritic activity [8, 9]. In the same study, the ethanol extract of CI (CIE) also exhibited anti-arthritic activity at a dose of 0.5 g/kg as a preliminary experiment. We have also reported the presence of oleanolic acid and scopoletin in CIE [10]. Oleanolic acid had anti-inflammatory activity in rats and mice [11] and anti-arthritic effect on adjuvant-induced chronic arthritis models [12]. Scopoletin also had anti-arthritic effects in rat adjuvant-induced arthritis models [13]. The present study was designed to investigate the potential therapeutic effect of CIE on mice with adjuvant induced joint arthritis, which is a model of autoimmunity diseases with great similarities to human rheumatoid arthritis [14].

## Methods

### Materials

Male Kunming mice weighing 25–30 g were purchased from Slac Laboratory Animal Co. Ltd. (Shanghai, China). All the procedures were approved by Animal Ethical Council of Nanjing University of Chinese Medicine. The mice were maintained under laboratory conditions at 25 °C under a normal 12 h/12 h light/dark cycle with humidity of 55% and fed with food and water ad libitum. The mice were allowed 7 days to adapt to the laboratory environment before experiments.

### Plant materials and preparation of extract

Samples of the dried root of CI were collected from Guangxi Province in October 2013 and identified by Prof. Jianwei Chen (Nanjing University of Chinese Medicine, Jiangsu, China). A voucher specimen was deposited at the herbarium of the Nanjing University of Chinese Medicine College of Pharmacy, Jiangsu (No. 102). The dried stem and root of CI (6.5 kg) was extracted by reflux method thrice with 95% ethanol. The ethanol filtrate was concentrated under reduced pressure at 55 °C by a vacuum rotary evaporator (RE-52A, Shanghai Yarong, China) and further dried in a vacuum drying oven (DZF-6090, Shanghai Jinghong, China) to yield a solid mass of weight 450 g (yield: 6.9%). The dried extract, CIE was freshly emulsified with 0.5% sodium carboxyl methyl cellulose (CMC-Na) and prepared with normal saline before use.

### Adjuvant-induced arthritis in mice and administration

All experimental procedures were conducted in conformity with institutional guidelines for the care and use of laboratory animals in China [Permit: SCXK (Shanghai) 2007–0005], and animal welfare and experimental procedures were strictly in accordance with the guide for the care and use of laboratory animals (National Research Council of USA, 1996). Adjuvant arthritis was induced on day 0 of the experiment by a single subcutaneous injection of 0.02 mL of complete Freund’s adjuvant (CFA) [containing 5.0 mg of dry, heat-killed *Mycobacterium tuberculosis* (strain H37Ra) per 1.0 mL sterile, non-metabolizable oils, Sigma-Aldrich, USA] into the plantar surface of right hind paw of the mice [15]. Equal amount of saline was injected to the left paw. This arthritis model, called adjuvant-induced arthritis (AIA) mice, has been widely used as a model of RA [16]. Another induction by an injection with 0.02 mL CFA in the base of tail was carried out 7 days after the first induction. 0.02 mL saline was injected in both paws and tail of vehicle control animals (10 normal mice). Since the second injection, AIA mice were divided into 3 groups [AIA model, CIE (0.4 g/kg) and CIE (0.8 g/kg) groups] randomly (with 10 mice in each group), and then the mice of CIE groups were received 0.4 and 0.8 g/kg of CIE by gastric intubation daily for 28 days. The mice of AIA model and vehicle control groups were given 0.1 mL/10 g of 0.5% CMC-Na instead.

### Adjuvant arthritis

#### Measurement of paw edema

Toxic symptoms, such as irritability, drowsiness, dyspnoea, ataxia and reflexes, and death during administration period in mice were observed and recorded. The disease recovery was evaluated by hind paws oedema index after sacrifice by cervical dislocation. The right hind paw of each mouse was dissected and weighed. The index of hind paws oedema was expressed as the ratio (mg/g) of hind paw weight versus body weight [9, 17, 18].

#### Arthritis score

The severity of arthritis was assessed by three independent observers. The mice were observed periodically for the verity of joint inflammation every 3 days after the second induction until sacrificed. The severity of arthritis was graded on a scale of 0–4 with the following criteria [19]: 0 = no edema or swelling, 1 = slight edema and limited erythema, 2 = slight edema and erythema from the ankle to the tarsal bone, 3 = moderate edema and erythema from the ankle to the tarsal bone, and 4 = edema and erythema from the ankle to the entire leg. The arthritis score for each mouse was the sum of severity in all four limbs (maximum 16 points for individual mice).

#### Measurement of MDA and ALP

The whole blood of mice in all the groups was collected through fossa orbitalis vein at day 28 after treatments. The serum was separated and divided into aliquots at 4 °C. The levels of malondialdehyde (MDA) and alkaline phosphatase (ALP) in serum were investigated by commercially available colorimetric assay kits (Jian Cheng Bioengineering Institute, China) according to the manufacturer instructions.

#### Spleen index determination

The mice of all the groups were sacrificed at day 28 after treatments, and the spleen were dissected and weighed. The index of spleen was calculated as the ratio (mg/g) of spleen wet weight versus body weight [9].

#### Histological examination

The specimens of knee joints were taken and fixed in 10% formalin and analyzed for pathological changes with hematoxylin and eosin. The severity of the arthritis was evaluated based on the changes in pathology, inflammation and erosion [20]. Morphological changes was graded on a scale of 0–4 with the following criteria: 0 = normal cartilage, articular cavity and synovial tissues, and no subcutaneous tissue inflammation, 1 = normal cartilage and articular cavity, slight synovial tissues thickening and inflammation, and slight subcutaneous tissue inflammation, 2 = normal cartilage and articular cavity, mild synovial tissues thickening and inflammation, and mild subcutaneous tissue inflammation, 3 = normal cartilage and articular cavity, moderate synovial tissues thickening and inflammation, and moderate subcutaneous tissue inflammation, 4 = normal articular cavity, damage cartilage, narrow joint space, severe synovial tissues thickening and inflammation, and severe subcutaneous tissue inflammation.

#### IL-1β and TNF-α expression by immunohistochemistry

Immunohistochemical detection of interleukin-1 beta (IL-1β) and tumor necrosis factor alpha (TNF-α) of the specimens of knee joints was performed by standard immunohistochemical techniques with primary antibodies against IL-1β and TNF-α (Bioworld, Shanghai, China), and horseradish peroxidase (HRP)-Polymer anti-Mouse/Rabbit IHC Kit (Mainxin-Bio, Fuzhou, China). Average density was measured with Motic Image Advanced 3.2 software (Motic China Group Co., LTD.).

### Statistical analysis

All results were presented as mean ± S.D. Data were analyzed by student’s *t*-test, two-way or one-way ANOVA followed by Dunnett’s *t*-test. Results were regarded statistically significant if the *P*-values were less than 0.05.

## Results

### Effect of CIE treatment on the macroscopic features of paw edema and spleen index

**Fig. 1:**
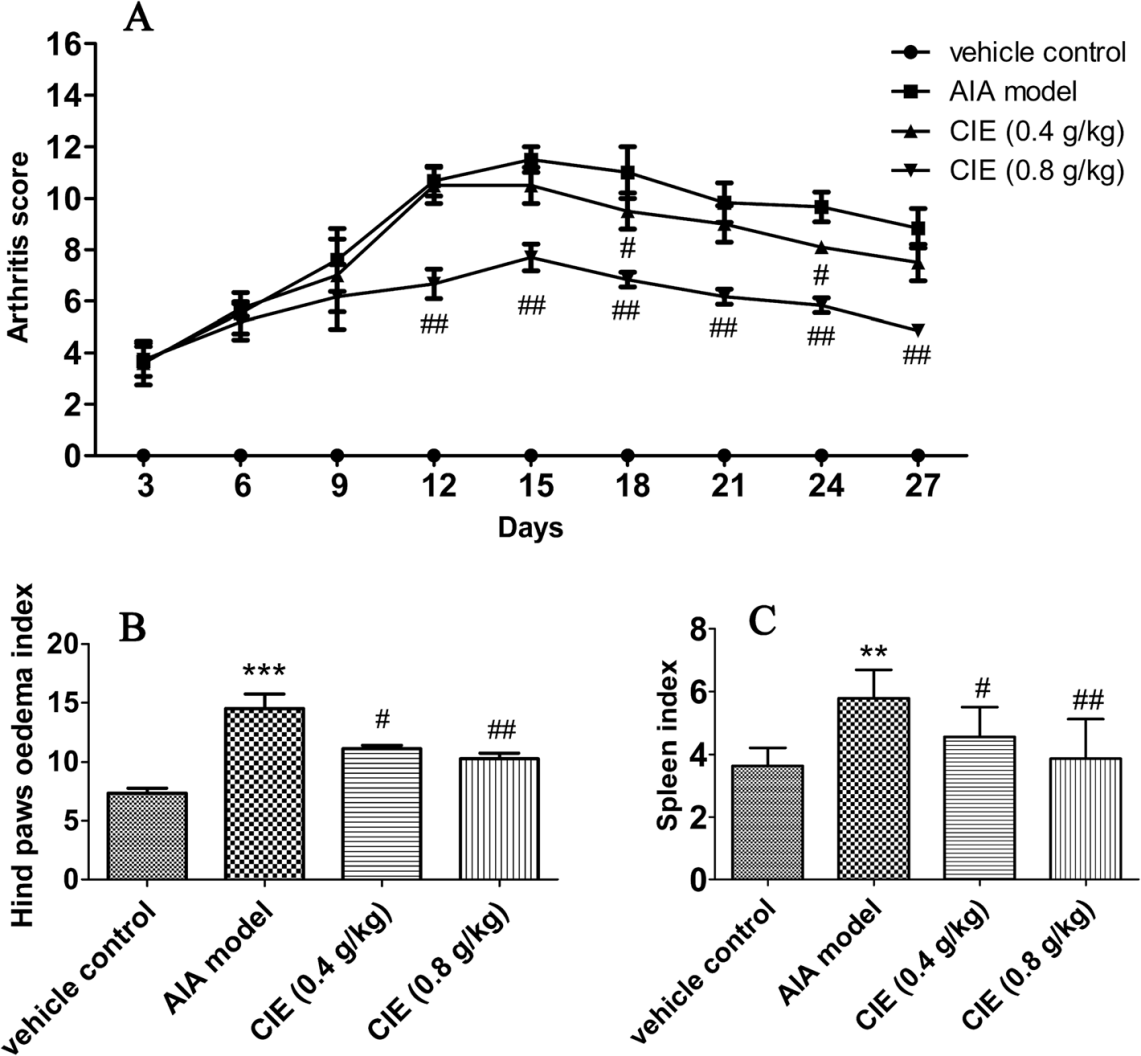
Anti-arthritic activity of CIE on arthritis score (**a**) and paw edema (**b**) in adjuvant-induced arthritis (AIA) mice. The spleen were promptly removed and weighed when the animals sacrificed after CIE was administered i.g. for 28 days. The spleen index (**c**) were expressed as the ratio (mg/g) of spleen wet weight versus body weight. The data was presented as mean ± S.D. of ten mice per group. ^**^ *p* < 0.01, and ^***^ *p* < 0.001 compared with the vehicle control group; ^#^ *p* < 0.05, and ^##^ *p* < 0.01 compared with the AIA model group

There was no sign of toxicity and mortality in mice treated with all tested CIE. It was observed that the body weight of mice increased gradually and the difference was not obvious in all test groups. As shown in Fig. 1a, the clinical scores of arthritis in AIA mice were dramatically increased compared to vehicle control mice, and were reduced by the treatment with CIE. The index of hind paw oedema (Fig. 1b) of CIE treated (0.4 g/kg and 0.8 g/kg) mice was lower (*P* < 0.05) than that of AIA mice significantly.

Compared to the vehicle control mice, the spleen index of the AIA mice was increased. Administration with CIE suppressed the hyperplasia dramatically. CIE treated (0.4 g/kg and 0.8 g/kg) mice showed a marked decrease (*P* < 0.05) of spleen index compared to that of the AIA mice (Fig. 1c).

### Effect of CIE treatment on histopathological changes

**Fig. 2:**
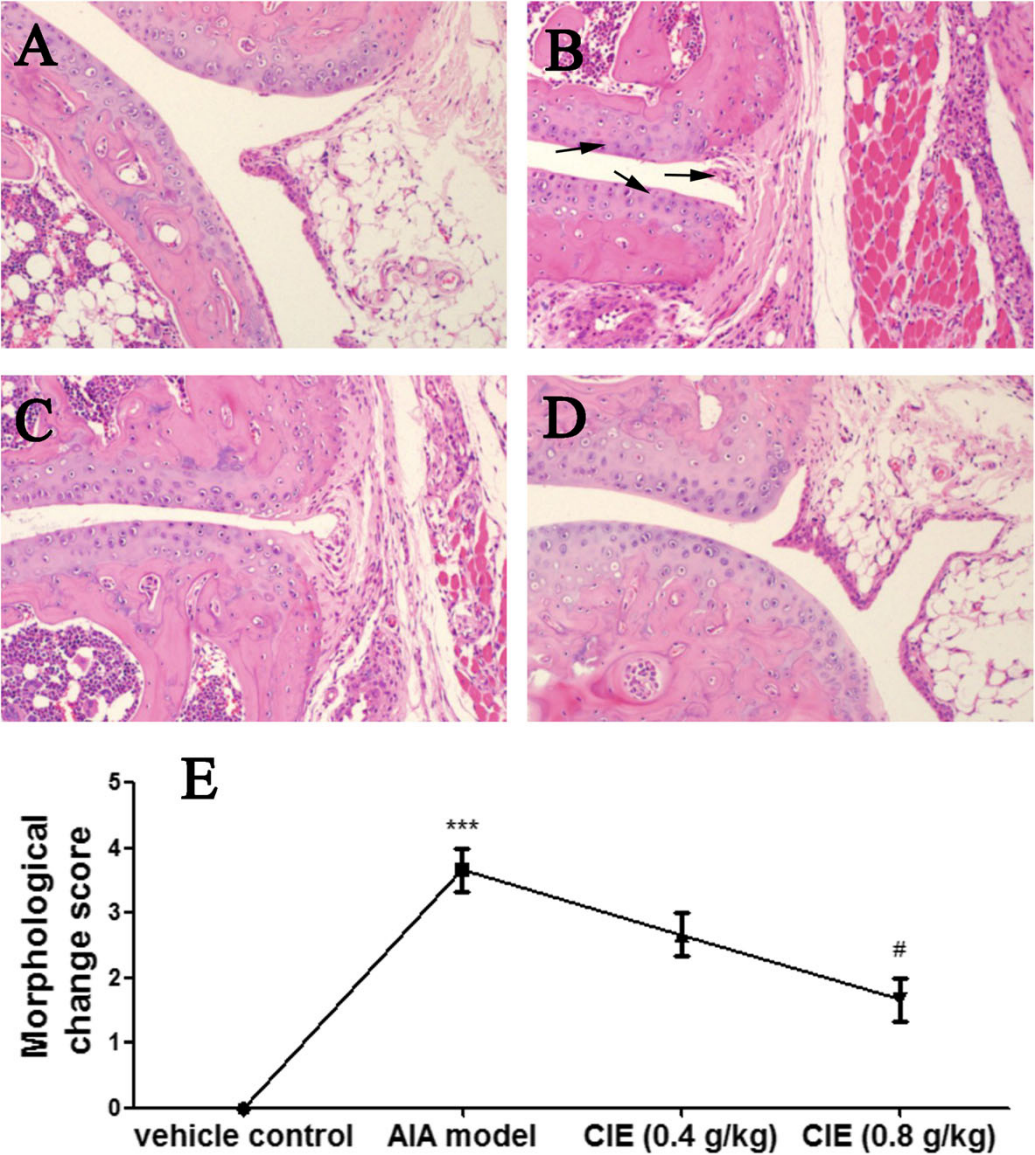
Effects of CIE on histopathological changes in the joints of AIA mice (original magnification 200×). **a** Vehicle control, (**b**) AIA model, (**c**) CIE (0.4 g/kg) and (**d**) CIE (0.8 g/kg). Representative sections from one rat are given for each group. All sections were stained with hematoxylin and eosin. Morphological changes (**e**) was graded on a scale of 0–4. The data was presented as mean ± S.D. of ten mice per group. ^***^ *p* < 0.001 compared with the vehicle control group; ^#^ *p* < 0.05 compared with the AIA model group

RA is well-characterized with synovial hyperplasia, pannus formation, cartilage and bone destruction in the joint. On day 28 after the primary treatment, the hind-paw joints of the mice were examined. As shown in Fig. 2b, in the AIA mice, the joints showed the infiltration of inflammatory cells, synovial hyperplasia, cartilage erosion and narrow joint space. Compared to vehicle control mice, morphological change score of AIA model mice was increased (*P* < 0.001) significantly (Fig. 2e). There was a statistically significant reduction (*P* < 0.05) in the morphological change score in the mice treated with 0.8 g/kg CIE, while a tendency towards a reduction in 0.4 g/kg CIE-treated mice with no significant difference.

### Modulatory effects of CIE on biochemical parameters of serum

**Fig. 3:**
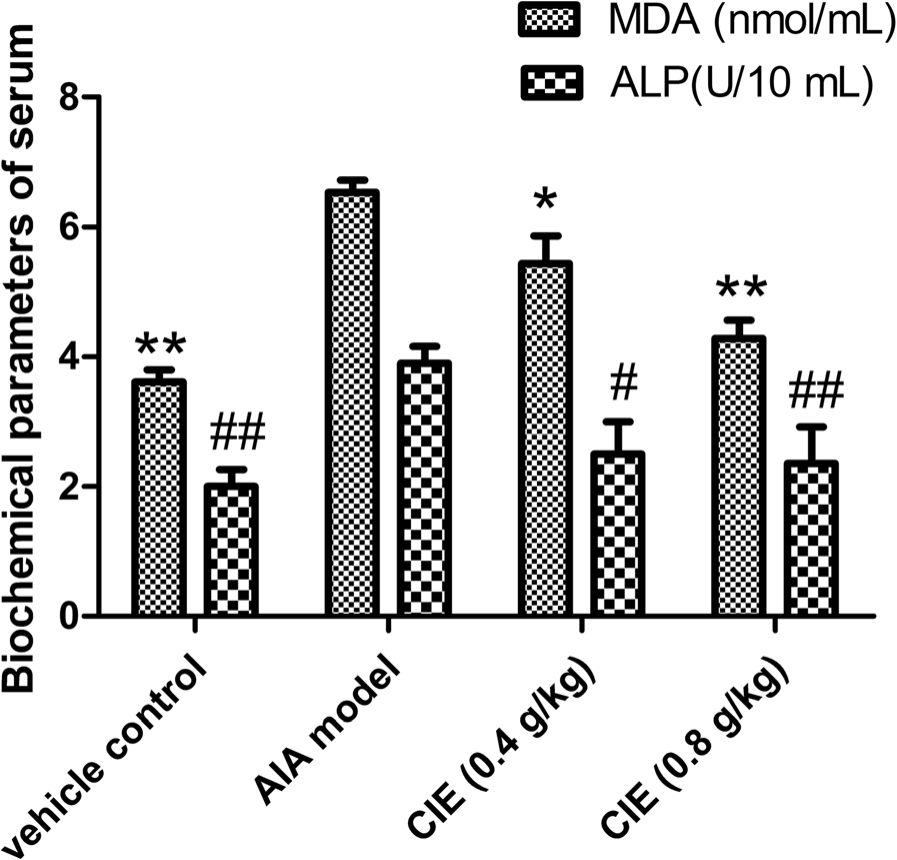
Biochemical parameters of serum. The data was presented as mean ± S.D. of ten mice per group. ^*, #^ *p* < 0.05 compared with the vehicle control group; ^**, ##^ *p* < 0.01 compared with the AIA model group

MDA and ALP levels were significantly higher (*P* < 0.01) in AIA model mice. The levels of MDA and ALP were significantly decreased (*P* < 0.01) compared with AIA model and CIE-treated mice (Fig. 3).

### Effect of CIE treatment on IL-1β and TNF-α production

**Fig. 4:**
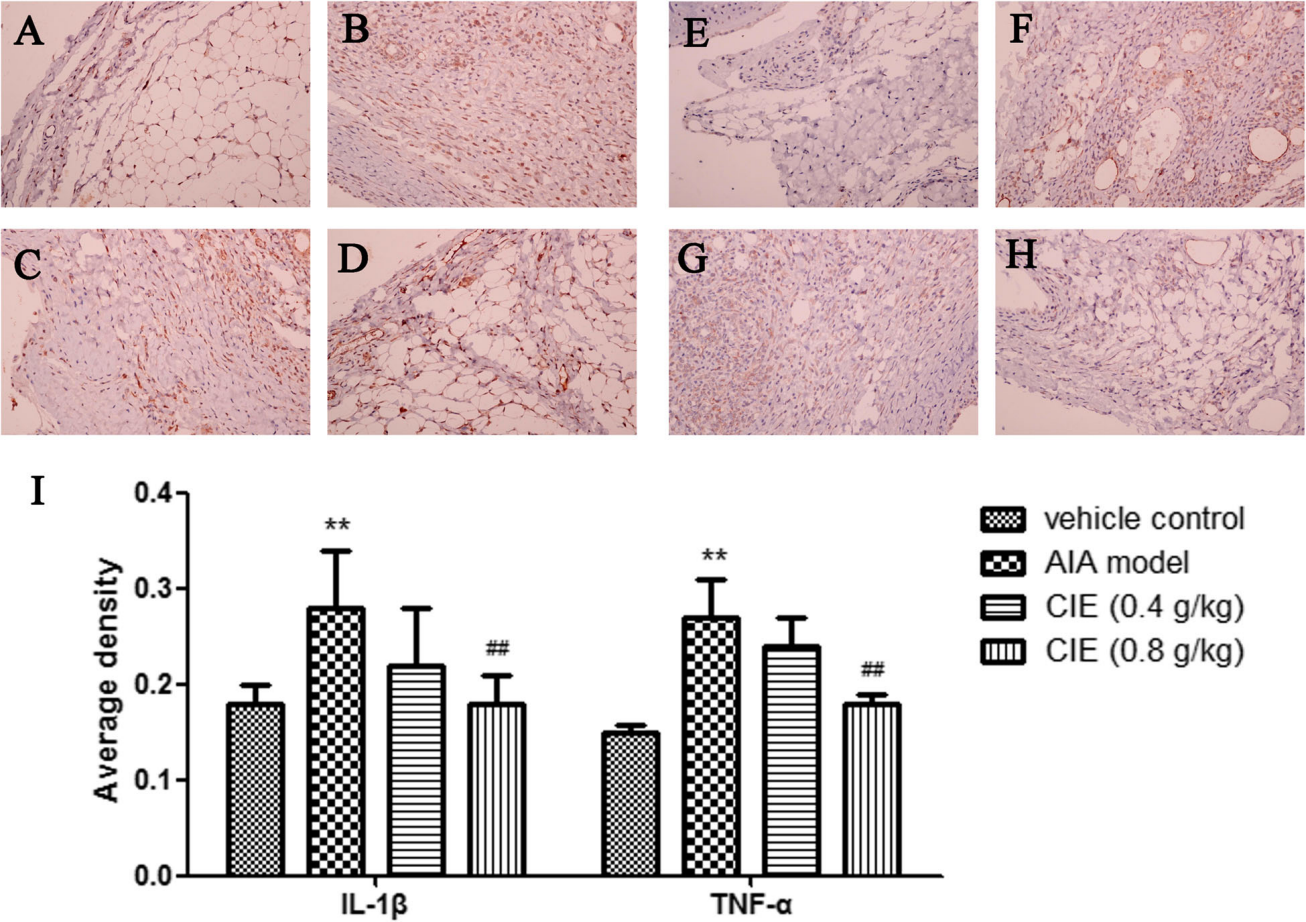
Immunohistochemistry of IL-1β (**a-d**) and TNF-α (**e-h**) in the knee joints taken from vehicle control mice (**a** and **e**), AIA model mice (**b** and **f**) and AIA mice after receiving repeated i.g. of 0.4 g/kg CIE (**c** and **g**) and 0.8 g/kg CIE (**d** and **h**) for 28 days. Magnification × 200. Tissues from three different mice in each group and 10 randomly selected areas from each slide were analyzed. Quantitative data (Mean ± S.D.) are presented using the average density values of the IL-1β and TNF-α positive regions. ^**^ *p* < 0.01 compared with the vehicle control group; ^##^ *p* < 0.01 compared with the AIA model group

IL-1β and TNF-α are reported to play critical roles in the pathogenesis of RA [21, 22]. Therefore, we investigated whether the therapeutic effects of CIE treatment were associated with changes in the frequencies of cells producing IL-1β or TNF-α in the arthritic joints by immunohistochemical staining. IL-1β- and TNF-α-positive cells were found distributed throughout the synovium, especially in synovial sublining regions (Fig. 4a-h). Compared to vehicle control group, the immunohistochemistry analysis showed that the expression of IL-1β and TNF-α was increased (*P* < 0.01) in AIA model mice (Fig. 4i). In addition, compared to AIA model group mice, there was a significant reduction (*P* < 0.01) of IL-1β and TNF-α expression in 0.8 g/kg CIE-treated mice.

## Discussion

In Traditional Chinese Medicine (TCM) theory, RA-related symptoms belong to the category of Bi Zheng, which can be manifested as arthralgia and dyskinesia of the joints and limbs due to an attack of the meridians of the limbs by wind, dampness, and heat or cold pathogens [23]. The use of NSAIDs and DMARDs cannot block the development and progress of RA, and has been impeded by their potential long-term side effects, toxicity and immunosuppression [24, 25]. It is important to search for new therapeutic drugs with greater efficiency and lower toxicity from a natural source [26]. So alternative therapies are popular among people with RA, and herbal products are receiving increasing public interest. CI has the functions of dispelling wind, eliminating dampness and relieving swelling pain, and is used as a folk remedy to treat RA, lumbocrural pain and foot edema in the south of China. However, to the best of our knowledge, there was no report about the anti-arthritic activity of CI.

The present study showed that CIE exhibited no toxicity and was well-tolerated by experimental animals at the tested doses (0.4 and 0.8 g/kg). Injection of oil based adjuvant under mice hind paw caused swelling and redness of paw and joints in the arthritic group of animals which persisted up to 15 days that signified the development of inflammation. Oral treatment with CIE significantly suppressed the increase of arthritis score and hind paws oedema index exhibited certain effect to treat RA, and showed significant protection against morphological changes of RA. These data indicated that CIE possessed a substantial anti-arthritic effect. Administration with CIE suppressed the increase of MDA in serum, which indicated a protective activity on membrane and restored free radical scavenging ability, and the down-regulation of ALP by CIE exhibited an effective anti-arthritic activity aiming at organo and bone protection.

In this study, CIE reduced the expression of IL-1β and TNF-α in knee joints synoviocytes of AIA model mice. It is believed that these inflammatory mediators play important roles in the pathogenesis of RA [27, 28] by degrading cartilage and bone, and then leading to the matrix degradation of tissue and aggravation of inflammation [29, 30]. Hence, the down-regulation of these inflammation mediators by CIE exhibits an effective anti-arthritic activity, which was beneficial for amelioration of AIA essentially.

## Conclusion

The results demonstrated that CIE effectively suppressed the pathological development, and possessed a substantial anti-arthritic activity in AIA mice. These beneficial effects were related to immumodepressive effects and the downregulation of inflammatory mediators, including IL-1β and TNF-α. Further studies on the mechanisms of action and active principles of CIE may contribute to the development of novel drugs for RA therapy.

## Abbreviations

AIA: Adjuvant-induced arthritis
ALP: Alkaline phosphatase
CFA: Complete Freund’s adjuvant
CI: *Claoxylon indicum* Hassk
CIE: Ethanol extract of *Claoxylon indicum*
IL-1β: Interleukin-1 beta
MDA: Malondialdehyde
MDARDs: Disease-modifying anti-rheumatic drugs
NSAIDs: Non-steroidal anti-inflammatory drugs
RA: Rheumatoid arthritis
SI: *Securidaca inappendiculata* Hassk
TNF-α: Tumor necrosis factor alpha
